# Catastrophic health expenditures in hospitalized patients with delta variant of COVID-19: A cross-sectional study

**DOI:** 10.34172/hpp.2023.09

**Published:** 2023-04-30

**Authors:** Zahra Gheinali, Esmaeil Moshiri, Masoumeh Ebrahimi Tavani, Mehdi Haghi, Farid Gharibi

**Affiliations:** ^1^Social Determinants of Health Research Center, Semnan University of Medical Sciences, Semnan, Iran; ^2^Quality Improvement, Monitoring and Evaluation Department, Center of Health Network Management, Deputy of Public Health, Ministry of Health & Medical Education, Tehran, Iran; ^3^Social Determinants of Health Research Center, School of Health and Nutrition, Lorestan University of Medical Sciences, Khorramabad, Iran

**Keywords:** Catastrophic, Health expenditures, COVID-19

## Abstract

**Background:** Financial protection of populations against healthcare costs is one of the fundamental responsibilities of governments. This study aimed to investigate the incidence of catastrophic health expenditures (CHE) and it’s affecting factors in hospitalized patients with delta variant of COVID-19.

**Methods:** In this cross-sectional study, we included 400 hospitalized COVID-19 patients at Kosar Hospital of Semnan in 2022, using a researcher-made checklist. Based on qualitative nature of the variables, chi-square test was used to investigate the statistical associations between the demographic/background characteristics and the incidence of CHE.

**Results:** On average, COVID-19 imposed 1833.43 USD direct medical costs per one hospitalized patient. The ratio of direct-medical costs to household’s non-food expenses was 2.35, and 61% (CI:±4.78%) of the patients were subject to CHE. Besides, residence place, basic insurance type, benefitting from supplementary insurance, suffering from underlying diseases, hospitalization in ICU, falling into a coma, facing pulmonary failure, and performing hemoperfusion had significant associations with CHE (*P*<0.05).

**Conclusion:** The incidence of CHE in hospitalized COVID-19 patients was undesirable, which may be due to geographical, economical, and occupational inequalities apart from the factors related to the severity of the disease. So, health policymakers should pay attention to the provision of proper financial risk protection policies to make the health insurance system more efficient and appropriate.

## Introduction

 Recently, all world countries experienced the most fatal epidemic in the past century, known as coronavirus disease 2019 (COVID-19). The disease spread in such a quick manner that within four months it resulted in three million confirmed infections, and two hundred thousand deaths in more than 209 countries.^1,2^ The incidence speed and scope of the destructive consequences of this disease was such that the World Health Organization (WHO) announced it as a full-blown pandemic in March 2020 and called the countries to fight against it all-out.^3^

 The most common symptoms of the disease include cough, fever, and shortness of breath, accompanied by general weakness, fatigue, dyspnea, muscle pain, sore throat, and loss of smell and taste.^4^ The patients with more severe conditions of the disease, such as breathing disorders, feeling pressure and pain in the chest, and loss of ability to speak and move require hospitalization and immediate clinical care. In addition to causing severe pulmonary problems, the disease can lead to serious cardiac complications, renal failure, acute hepatitis, hemoptysis, dyspnea, and leukopenia.^4,5^

 The high prevalence of COVID-19, the remarkable rate of patients with severe conditions requiring hospitalization, and the need for various costly diagnostic and treatment care, especially in the intensive care unit (ICU) and cardiac care unit (CCU), caused COVID-19 to be known as a costly disease for communities, especially in developing countries.^5-7^ Hence, it is crucial to study the imposed costs on patients, insurance companies, and the health system. In a previous study, the annual imposed costs of COVID-19 on Medicare insurance was estimated to be more than 6.3 billion dollars, 92.6% of which were allocated to the hospitalized patients.^8^ The cost of hospitalization per patient was also estimated at 21 752 dollars.^8^ In Turkey, the annual imposed cost on the health system due to the outbreak of COVID-19 was more than 1.2 billion dollars, which was equal to 2% of the total annual budget of the health system and 0.8 thousandth of the country’s gross domestic product (GDP).^9^

 Previous studies have only considered the costs on health insurance (as a third payer), health system, and society, and no study has yet assessed the imposed costs on patients with COVID-19, worldwide. Estimating the catastrophic health expenditures (CHE) indicator is one of the most critical actions to assess the imposed costs from the perspective of patients and their households, which can be helpful in indicating the financial supports provided to patients in different societies against health costs, and implementing promotional interventions, particularly in developing countries.^10^ CHE is calculated based on the amounts paid to the health care provider(s) by patients and their families, which is also referred to as the out-of-pocket payment and direct medical costs.^11^ In this study, we aimed to investigate the incidence of CHE in hospitalized patients suffering from COVID-19.

## Martials and Methods

###  Type of study and participants

 This cross-sectional study was carried out on 400 patients with delta variant of COVID-19 hospitalized in Kosar Hospital of Semnan, Iran from September to December 2022. The inclusion criteria were being admitted to the hospital, and passing at least one month from the time of discharge. We considered ‘hospitalization’ as an inclusion criterion, because many patients have either no symptom or mild symptoms with no need to receive care. Also, the most of patients with symptoms receive cheap outpatient care and do not require hospitalization. Nevertheless, the hospitalized patients are hospitalized for more than two weeks on average and have a longer recuperation period and costs. “Passing at least one month from discharge” was another inclusion criterion, because the disease has a long recuperation period with especially respiratory complications and excessive fatigue, which may result in low levels of patients’ participation in the study. Also, “passing at least one month from discharge” minimizes the possibility of recall bias and the risk of transmission of disease to the interviewers. We excluded all patients who died, because the probability of participation by the family members was very low. Also, many of them might be unaware of the demographic and background characteristics of the deceased patients.

 Semnan is a province in central Iran with a population of more than 700 000 people and an area of 96 816 square kilometers. Semnan is an industrial province due to its short distance from Tehran (the capital city), Karaj (a megapolitan city), and Northern provinces (as touristic area of Iran). Because of low population and appropriate job opportunities, Semnan is one of the best Iranian provinces in terms of economic status, especially in misery index (an economic indicator calculated by adding the seasonal adjusted unemployment rate to the annual inflation rate).

###  Sample size and sampling method

 Sample size was calculated to be 384 (attrition rate of 5% = 400), based on the following formula. Due to the lack of similar studies, the standard deviation was estimated from a pilot study on patients.

$$n\;=\;{\left[\frac{zs}{d}\right]}^{2}\;={\left[\frac{1.96\;*\;20000000}{20000000}\right]}^{2}\;=\;384.16$$

The sampling method was simple random sampling from the list of patients. We collected the data related to direct medical costs imposed on COVID-19 patients by examining the medical records of the patients and patients’ interview. We also conducted interviews to collect other data related to demographic and background characteristics, direct non-medical costs, indirect costs, as well as the patients’ views on the imposed costs.

###  Study tools

 We developed a checklist to obtain the required data. All components related to the imposed health costs and demographic/background characteristics were initially identified after a literature review, and interviewing the specialists and the patients. Then, the extracted components were considered as the items for the development of the initial checklist. Subsequently, the checklist was evaluated and confirmed by ten experts. To assess content validity, all the items of the checklist were examined from the perspective of experts based on the four indicators of necessity, relevance, transparency, and simplicity; content validity ratio (CVR) and content validity index (CVI) was calculated based on below formula^12^:

$$CVR\;/\;CVI\;=\;\frac{nE\;-\;\frac{N}{2}}{\frac{N}{2}}$$

Where *nE* was the number of experts choosing two positive spectrum choices and *N* indicated the total number of experts. Due to the participation of ten experts in this stage, the acceptance score of 0.62 was the basis for approving/disapproving the items to be included in the checklist.^12^ The final tool was a 75-item checklist, including 23 items related to demographic and background characteristics, 42 items related to the cost information of the disease, four items related to the income status of the patients and their family, and six items related to the patients’ views on the negative consequences of the disease costs. The validity of the checklist was confirmed by the experts. The final scores for CVR and CVI indicators were 0.88 and 0.92, respectively.

###  Main variable definition

 The concept of CHE implies that the cost of health care is higher than the capacity to pay (CTP) of patients and their families. Also, if the direct health costs paid by patients and their families exceed 40% of the non-food expenses of the household, there would be a CHE occurrence.The main data examined in the study included the types of direct medical costs imposed on patients and their families in the form of out-of-pocket payments and at the time of receiving COVID-19-related health care (diagnosis, treatment, and rehabilitation). Therefore, the following formula was used to calculate the CHE^13^:

* CHE=HealthE>CTP*

* HealthE: Direct medical costs based on out-of pocket payments*

* CTP: Expenses of equal and more than 40% of non-food costs of households for healthcare*

* Subsequently:*

* CHE=Direct Medical costs≥40% household non-food costs*

 To calculate CHE, the information related to the imposed health costs on the patients and their households during a defined period was calculated. Then, to determine the level of CHE occurrence, this amount was compared to the household income during the considered period. Due to the lack of comprehensive health records for the patients, the amount and type of mentioned expenses were collected from the patients through self-report, and the accuracy of the provided information was compared with their hospital records. As mentioned above, the main criterion for the incidence of CHE in a patient and his/her family was the amount of out-of-pocket payments reaching a certain threshold level of his/her income (capacity to pay). Based on the recommendations of the WHO and the most of similar studies, this threshold was considered as 40% of non-food expenses of a family.^11^ Since the amount of out-of-pocket payment was calculated solely based on the direct medical costs (diagnosis, treatment, and rehabilitation) and the direct non-medical costs (such as the cost of adapting [immunizing] the home and commuting to care centers), the indirect costs (such as absence in the workplace, and intangible costs such as the imposed pain on the patient and his/her family) were not considered in the study.

 We investigated different demographic and underlying variables such as age, gender, employment, and education status of the patient, having basic and supplementary health insurance, the pattern of receiving healthcare services (private and public centers), place of residence, suffering from a comorbidity, the type and severity of side effects, their association with the amount of imposed expenditure on patient and his/her family, and the catastrophic nature of these expenditures.

###  Data analysis 

 Missing data in the patients’ checklist were completed by contacting the respondents again. If a respondent was not available or did not answer the questions related to the missing data, his/her associated checklist was excluded from data analysis. In this case, the patient with the next assigned code was included in the study.

 The data analysis was first descriptive and then analytical. The results of descriptive studies were considered and reported for qualitative variables as frequency (percentage) and for quantitative variables as mean (standard deviation). Primarily, the incurred direct medical costs were calculated in Iranian Rials (IRR) and then changed to US dollars (USD) using the exchange rate announced by the central bank of Iran (1 USD = 42 000 IRR).

 Based on the qualitative nature of the variables, the chi-square test was used to investigate the statistical associations between demographic and background variables and the incidence of CHE. All analyses were performed using the SPSS v. 19, and *P* < 0.05 was considered as significant.

## Results

 Mean age of participants was 56.20 (± 59.77) years. The gender ratio of patients was almost equal and most of them were married. The most of patients were housekeepers and self-employed. A majority of the patients were urban dwellers and lived in the center of the province (Table 1).

**Table 1:** Demographic characteristics of the patients participated in the study (n = 384)

**Demographic Variables**	**Category**	**Frequency**	**Percent**
Age	1 to 20 years old	6	1.50
	21 to 40 years old	106	26.50
	41 to 60 years old	161	40.25
	61 years and older	127	31.75
Gender	Male	188	47.00
	Female	212	53.00
Marital status	Single	45	11.25
	Married	344	86.00
	Divorced	11	2.75
Educational status	Illiterate	62	15.50
	Below high school diploma	71	17.75
	High school diploma	86	21.50
	Associate and bachelor degrees	159	39.75
	Master degree	22	5.50
Occupational status	Office employee	78	19.50
	Worker	10	2.50
	Self-employed	83	20.75
	Housekeeper	142	35.50
	Retired	73	18.25
	Student	12	3.00
	Unemployed	2	0.50
Residency	Center of province	383	95.75
	Other places of province	14	3.50
	Outside of province	3	0.75
Living place	Urban dwellers	364	90.50
	Rural dwellers	18	9.50

 As there is shown in Table 2, almost all patients had basic health insurance, however, less than a third of patients had supplementary health insurance. A majority of patients received the needed care only from the governmental sector. Mostly, had a background disease, such as cardiac disease, hypertension, and diabetes. Most of the patients with a background disease reported that their condition was under control before contracting COVID-19. Although the majority of patients did not have continuous sports activity before the disease, almost half of them reported that they had enough physical activity due to the nature of their jobs. Less than a quarter of the patients needed hospitalization in the ICU. The mean hospitalization periods of the patients requiring hospitalization in the ward, ICU, and hospitalization in both the ward and ICU were 4.10, 1.62, and 5.69 days, respectively. Only 7% of the patients had experienced coma (0.28 days per patient), however, all had suffered serious side effects, especially respiratory failure and cardiac problems due to the COVID-19 infection (Table 2).

**Table 2:** Background characteristics of the participants (n = 384)

**Background Variables**	**Category**	**Frequency**	**%**
**Benefiting basic insurance**		390	97.50
Type of basic insurance	Social security	249	63.80
	Treatment services	92	23.50
	Armed forced	35	8.70
	Other	16	4.10
**Benefiting supplementary insurance**		119	29.75
Place of care	Governmental center	340	85.00
	Integration of private and public centers	60	15.00
**Suffering from a background disease**		346	86.50
Type of background disease	Cardiac problems	162	40.50
	Hypertension	165	41.25
	Pulmonary diseases	68	17.00
	Asthma	30	7.50
	Diabetes	118	29.50
	Cancer	4	1.00
	Immune system defect	29	7.25
	Obesity	24	6.00
Having background disease under control	Yes	331	95.66
	Somewhat	15	4.34
Continuous exercise before the infection	Yes	48	12.00
	No	250	62.50
	Somewhat	102	25.50
Having occupational physical activity	Yes	46	11.50
	No	165	41.25
	Somewhat	189	47.25
Hospitalization	In the ward	368	92.00
	In the ICU	95	23.75
	Both ICU and ward	62	15.50
**Fall into a coma**		28	7.00
Serious side effects		396	99.00
Type of side effects	Cardiac problem	271	67.75
	Pulmonary problem	384	96.00
	Renal problem	25	6.25
	Need for hemoperfusion	22	5.50

 Examining the direct medical costs indicated that the largest part of the imposed diagnostic costs on the patients was the diagnostic costs associated to the side effects of the disease and laboratory services, respectively (Table 3). Also, the largest part of the medical services’ costs was allocated to hemoperfusion and medication preparation, respectively. Although hemoperfusion had only been performed for 5.5% of the patients, it imposed the highest treatment costs (Table 3). In total, 1833.43 USD (77 004 080 IRR) were allocated to direct medical costs imposed on each hospitalized COVID-19 patient.

**Table 3:** Direct medical costs imposed on the patients participated in the study (n = 384)

**Domain**	**Type of costs**	**Minimum**		**Maximum**		**Mean (standard deviation)**	
		**IRR**	**USD**	**IRR**	**USD**	**IRR**	**USD**
Diagnosing services	Laboratory services	0	0	30000000	714.28	1858300 (± 21222310)	44.24 (± 505.29)
	Radiology	0	0	1470000	35	10050 (± 107920)	0.24 (± 2.57)
	CT scan	0	0	1380000	32.85	107250 (± 199890)	2.55 (± 4.76)
	Other diagnosing costs	0	0	980000000	23333.33	6857150 (± 74381400)	163.26 (± 1770.98)
	Sum of diagnosing expenditures	0	0	981260000	23363.33	8798470 (± 77084110)	209.48 (± 1835.33)
Treatment services	Visiting general physician	0	0	1000000	23.80	114200 (± 1067900)	2.71 (± 25.42)
	Visiting specialist physician	0	0	9900000	235.71	452170 (± 1067900)	10.76 (± 25.42)
	Sum of visiting expenditures	0	0	10400000	247.61	566370 (± 1205220)	13.48 (± 28.69)
	Hospitalization services	0	0	250000000	5952.38	4520730 (± 19353590)	107.63 (± 460.79)
	Medication	0	0	380000000	9047.61	27585000 (± 41162380)	656.78 (± 980.05)
	Traditional treatments	0	0	5000000	119.04	119090 (± 547110)	2.83 (± 13.02)
	Treatment cares at home	0	0	23000000	547.61	565000 (± 2500910)	13.45 (± 59.54)
	Hemoperfusion	0	0	1300000000	30952.38	35025120 (± 170903340)	83393 (± 4069.12)
	Unofficial payments	0	0	0	0	0	0
	Sum of treatment services (apart from visits)	0	0	1680090000	40002.14	67639230 (± 200349660)	1610.45 (± 4770.23)
Sum of expenditures		0		1980090000	47145	77004080 (± 22819737)	1833.43 (± 543.32)

 The mean monthly income of the patients’ families was about 1651.07 USD (69 345 000 IRR), within which 779.52 USD (32 740 000 IRR) was spent on non-food expenses. Calculating the incidence of CHE in the examined patients showed that the mean ratio of direct medical costs to non-food expenses of the families was 2.35, which was six times greater than its normal amount (less than 0.4 or 40%). Accordingly, 61% (CI: ± 4.78%) of the examined patients were subject to CHE.

 As the patients reported, 39% had given up and 34.5% had delay in receiving the necessary services recommended by the doctors, due to their high costs. Also, 38.5% attempted to receive cheaper and/or lower quality health care. Meanwhile, 26.5% received financial assistance from their friends and charitable institutions to cover their treatment costs, and 48.5% obtained loans or borrowed money from banks and friends. Generally, 60.5% reported that they faced serious problems to meet their medical expenses.

 As reported in Table 4, among demographic and background characteristics, the place of residency, type of basic insurance, benefitting supplementary insurance, suffering from an background disease, hospitalization in ICU, combined hospitalization in both the ward and ICU, falling into coma, respiratory failure, and hemoperfusion had a statistically significant association with CHE (*P* < 0.05). According to the results, the incidence of CHE in the patients living in urban areas was 37% higher than those living in rural areas. CHE in the patients with supplementary insurance was 73% lower than those without the insurance. Furthermore, in patients hospitalized in ICU, the CHE was 20% higher than ICU non-hospitalized patients. Also, in patients with a history of coma, the CHE was 34% higher than those without such history. In individuals with a history of hemoperfusion, CHE was 42% higher compared to those without hemoperfusion experience (Table 4).

**Table 4:** Statistical association between demographic/background characteristics and CHE incidence

**Variable**	**Category**	**Incidence (CI) as %**	***P*** value*
Residency	Urban	58 (± 4.83)	0.002
	Rural	95 (± 2.13)	
Type of insurance	Social security	65 (± 4.67)	< 0.001
	Treatment services	72 (± 4.40)	
	Army forces	23 (± 4.12)	
	Others (banks, petroleum company, etc)	0 (0)	
Benefiting supplementary insurance	Yes	10 (± 2.94)	< 0.001
	No	83 (± 3.68)	
Suffering from a background disease	Yes	58 (± 4.83)	0.020
	No	72 (± 4.40)	
Hospitalization in an ICU	Yes	65 (± 4.67)	0.224
	No	45 (± 4.87)	
Hospitalization in both the ward and ICU	Yes	84 (± 3.59)	0.003
	No	57 (± 4.85)	
Falling into a coma	Yes	93 (± 2.50)	0.010
	No	59 (± 4.82)	
Respiratory failure	Yes	59 (± 4.82)	0.024
	No	0 (0)	
Hemoperfusion	Yes	100 (0)	0.008
	No	58 (± 4.83)	

* Chi-square test.

## Discussion

 In this study, we investigated the incidence of CHE in hospitalized patients suffering from COVID-19. According to the results, contracting COVID-19 imposed a mean of 1833.43 USD (77 004 080 IRR) direct medical costs on each Iranian hospitalized patient. Li et al., reported the total (direct and indirect) incurred costs on patients with COVID-19 in china to be 6827 USD.^14^ Bartsch et al in the United States also estimated the direct medical costs of the disease as 3045 USD.^15^ In Iran, Ghaffari Darab et al reported the direct medical costs as 3755 USD, ^16^ and Nakhaei et al estimated the direct (medical and non-medical) costs as 3362 USD.^17^

 In our study, the largest part of the imposed costs was due to hemoperfusion and medication preparation, respectively, in a way that these two components accounted for 81% of the direct medical costs (out-of-pocket payments). Since most of the used equipment and medicines in the treatment of Iranian COVID-19 patients are imported, they impose a high cost on the health system. If such equipment and medicines were produced inside the country, their cost would be greatly reduced for both the patients and the health system. Also, the possibility of lack of equipment and medicines during conditions like COVID-19 pandemic around the world is very high. Such shortages may be due to the governing principles of a competitive economy, which can lead to a sharp increase in market prices due to high demand and insufficient supply. However, we should not ignore the strong role of political, economic, and health sanctions that severely limit the access of some countries to necessary equipment and medicines and/or their production technology.^18^

 Although hemoperfusion for the patients with severe blood oxygen depletion was performed only in 5.5% of the patients, it accounted for half of the total direct medical costs. Hemoperfusion is not recommended as an essential care in any of the clinical protocols and guidelines, and thus only a small number of specialists, based on their personal experience, perform and recommend it. Therefore, absolute adherence to reliable scientific evidence can have a significant role in reducing the imposed costs on the patients. In the study conducted by Nakhaei et al., age, having supplemental health insurance, hospitalization in ICU and death were the factors associated to the amount of incurred cost on the patients.^17^

 In our study, although the mean monthly income of the patients’ families was 1651.07 USD (69 345 000 IRR), half of the amount was spent on non-food expenses, and an amount greater than the total income (1833.43 USD equal to 77 004 080 IRR) was spent on health care. We also found that the mean ratio of direct medical costs to non-food expenses of families was 2.35, which was six times greater than its normal amount (less than 0.4 or 40%). Accordingly, 61% of the examined patients were subject to CHE. It should be noted that this study was conducted in a public hospital affiliated to the Ministry of Health, which strictly adheres to governmental tariffs. Certainly, conducting a similar study in private hospitals might show a higher incidence of CHE.

 As far as we know, no study has yet been published to evaluate the CHE in patients with COVID-19 infection. Few studies evaluated the COVID-19-related costs mostly based on the imposed costs on the health system and health insurance organizations (as third-party payers), and not from the perspective of the patients and their families. So, these studies cannot judge the social protection of citizens against the COVID-19 costs.

 Studies on the incidence of CHE in other diseases showed that CHE in cancer patients was estimated as follows: 27%,^19^ 43%,^20^ and 95%^21^ in China, 75% in Ethiopia,^22^ 48% in Malaysia,^23^ 50% in South Korea,^24^ and 79% in India.^25^ In addition, the incidence of CHE in patients with heart diseases was reported to be 54%^26^ and 84%^27^ in India, and 55% in Iran.^28^ Also, CHE in patients suffering from hypertension was calculated to be 24%^29^ and 14%^30^ in China. Likewise, in a study in Iran, the CHE imposed on multiple sclerosis patients was estimated to be 54%.^10^ The results of another study indicated that 6.2% of the families with a neoplasm patient, 12.9% of the families with a member suffering from brain diseases, 9.6% of the families with a diabetes patient, 7% of the families with a member suffering from arthritis, 23.6% of the patients with kidney disease, 7.3% of the patients with hypertension, and 11.7% of the families with a member suffering from ischemic heart diseases faced CHE.^31^

 Although literature showed that CHE often occurs in chronic diseases, especially diseases that require surgery and complex and expensive treatments, infectious diseases have also the potential for the incidence of CHE. Accordingly, the incidence of CHE was estimated to be 52% in the patients with tuberculosis in China,^32^ and 33% in the hospitalized patients with gastroenteritis in Malaysia.^33^ Even mental-psychological disorders such as depression can make 24% of the patients and their family experience CHE.^34^

 Our results showed that living in urban areas, having basic insurance of specific types (army forces, banks, petroleum company, etc), and having supplementary insurance strongly affected the incidence of CHE. These findings indicate the role of geographical, occupational, and financial inequalities in access to health care needed by COVID-19 patients. We also found that 60% of people with basic insurance experience CHE, which shows poor and ineffective coverage of this insurance even in the governmental centers. Another study on multiple sclerosis patients showed that the geographical, economical and job-related factors affected the occurrence of CHE, significantly.^18^

 The lower probability of CHE incidence in the urban population can be due to financial (economical) and geographical (physical) issues and even better cultural access to the needed care, which may lead to earlier and better follow-up of the disease, and receiving timely and complete appropriate treatments. Another reason might be the higher mean income of urban dwellers, as their jobs are more specialized, lucrative, and permanent compared to the seasonal jobs of rural participants. Obviously, the type of basic health insurance, which is an occupational advantage, can also be considered as a serious source for inequality. As previously noted, the factors like patients’ age, supplemental health insurance, hospitalization in ICU and death (as disease severity factors) were associated to the amount of incurred cost on patients.^17^ Similar studies have also shown that benefiting from basic health insurance,^35-37^ having supplementary health insurance,^10,38^ and having certain types of insurance with specific advantages^27^ can lead to a significant reduction in the probability of CHE incidence. Also, living conditions of the patients and their families, being close to the central parts of the province due to the possibility of benefiting from treatment facilities,^10,38^ and the area of residence (rural or urban)^19,37,39^ had significant effects on the incidence of CHE.

 In terms of the severity of the disease and its negative consequences, we found that the hospitalized patients in the ICU, patients with a history of being hospitalized in both the ICU and ward, patients experiencing coma, those with respiratory failure (as one of the side effects of COVID-19), and patients with a history of hemoperfusion due to a severe deplete in blood oxygen faced significantly higher health costs than other patients. Accordingly, the patients with a more severe disease need more supportive health insurances. In terms of hemoperfusion, which significantly increases the probability of CHE, we found that 100% of the patients receiving this service experienced CHE, despite the fact that performing this procedure as a necessary action is not confirmed by treatment protocols. Some previous studies identified the severity of the disease and subsequently the need for the patient to benefit from more complex, specialized, and expensive facilities as the factors affecting CHE.^10,39^

 One of the limitations of this study was the impossibility of data collection for deceased cases. If it was possible to benefit from the data of those who suffered a more severe disease and possibly experienced a higher cost, the CHE would be higher than the current amount. Another limitation was the impossibility of collecting data from private hospitals to compare with governmental centers, which could have produced more interesting results, because generally hospitalized patients in private hospitals have a much higher cost and are more likely to face CHE. The last limitation was the collection of data in the period from September to December 2021 while Iran was at the peak of the Delta strain. Therefore, the results of this study cannot be attributed to other strains and their peaks.

 Based on the results of this study, it is recommended that the country officials attempt to increase the value of national currency, provide better access to equipment and medicines, and develop the production technology related to equipment and medicines. To do so, some strategies might be as follows: paying attention to the internal capacities to produce necessary equipment and medicines by strengthening the knowledge-based companies, developing treatment protocols and guidelines for COVID-19 and its various strains, monitoring the unconditional compliance of health centers with the developed treatment protocols, paying more attention to COVID-19 vaccination and encouraging citizens to inject the vaccine and its boosters on time, creating a more efficient screening mechanism and timely diagnosis of the disease, creating more efficient insurance mechanisms, especially with emphasis on rural patients who lack financial ability, and the expansion of insurance coverage for acute and serious cases of diseases to reduce exposure to CHE in COVID-19 patients. Further studies in private hospitals are required during different peaks and strains of the disease to assess the current situation and the impact of possible implemented interventions.

## Conclusion

 Our results showed that in contrast to the idea that the incidence of CHE is often attributed to chronic diseases or complex surgeries, infectious diseases such as COVID-19 can lead to CHE if the patient is hospitalized. Also, serious geographical, financial, and occupational inequalities might lead to the incidence of CHE. As the existing insurance systems fail to provide adequate support for the complex and severe cases of this disease, it seems to be essential to develop national-level interventions aiming at improving the social protection systems of the patients. The results of present study can help health policymakers and those in charge of state health insurance to understand the importance of the CHE issue and plan for the development of appropriate interventions.

## Acknowledgments

 We thank all the patients for their participation in this study. We also acknowledge the staff of Kosar Hospital of Semnan for providing the necessities for the research and the Vice-Chancellor for Research and Technology of Semnan University of Medical Sciences for financial support.

## Competing Interests

 The authors declare that they have no conflict of interest.

## Ethical Approval

 All patients were free to accept or refuse to participate in the study. An informed consent was obtained from all participants, and they were assured that the results would be published in an anonymous manner. The privacy of the study participants was respected, and the participants were assured that the obtained results would be used only for research aims. Also, the Ethics Committee of Semnan University of Medical Sciences approved the research protocol (code: IR.SEMUMS.REC.1400.204).

## Funding

 The study was founded by Semnan University of Medical Sciences, Iran.
